# Symmetry reduction due to gallium substitution in the garnet Li_6.43(2)_Ga_0.52(3)_La_2.67(4)_Zr_2_O_12_

**DOI:** 10.1107/S2056989016001924

**Published:** 2016-02-06

**Authors:** Lars Robben, Elena Merzlyakova, Paul Heitjans, Thorsten M. Gesing

**Affiliations:** a Chemische Kristallographie fester Stoffe, Institut für Anorganische Chemie und Kristallographie, FB02, Leobener Strasse/NW2, and MAPEX Center for Materials and Processes, Universität Bremen, Bibliotheksstrasse 1, 28359 Bremen, Germany; b Institut für Physikalische Chemie und Elektrochemie, Leibniz Universität Hannover, Callinstrasse 3-3a, D-30167 Hannover, Germany

**Keywords:** crystal structure, garnet, lithium lanthanum zirconate (LLZO), symmetry reduction

## Abstract

Single-crystal structure refinements on lithium lanthanum zirconate (LLZO; Li_7_La_3_Zr_2_O_12_) substituted with gallium were successfully carried out in the cubic symmetry space group *I* 3*d*. Gallium was found on two lithium sites as well as on the lanthanum position. Due to the structural distortion of the resulting Li_6.43(2)_Ga_0.52(3)_La_2.67(4)_Zr_2_O_12_ (Ga–LLZO) single crystals, a reduction of the LLZO cubic garnet symmetry from *I*a *d* to *I* 3*d* was necessary, which could hardly be analysed from X-ray powder diffraction data.

## Abstract 2

Gallium-substituted lithium lanthanum zirconate (LLZO; Li_6.62_La_2.65_Ga_0.49_Zr_2_O_12_) belongs to the family of garnets and shows a reduction of the symmetry to space group *I* 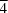
3*d* compared to *I*a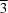 *d* typically observed for these structures.

## Chemical context   

Garnets can be described with the ideal formula *A*_3_ *B*_2_(*X*O_4_)_3_ in space group *I*a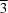 *d*, with different coordination polyhedra of the respective elements with oxygen, resulting in a distorted cube for *A* (*e.g.* Ca), an octa­hedron for *B* (*e.g.* Al) and a tetra­hedron for *X* (*e.g.* Si). The variability of the elements on the crystallographic sites (thereby keeping the high symmetry) gives rise to inter­esting material properties like ferrimagnetism (Geller, 1967 ▸). In recent years, garnet-type compounds containing Li have gained considerable inter­est as promising electrolyte materials for all-solid-state Li-ion batteries. The so-called ‘Li-stuffed’ garnets, which contain more Li than available on tetra­hedral sites (*X*), meaning that excess Li occupies other sites as well, show an increase in Li-ion mobility. An exhaustive overview of these compounds was recently given by Thangadurai *et al.* (2014 ▸). The garnet-type fast lithium ion conductor Li_7_La_3_Zr_2_O_12_, abbreviated as LLZO, is such an ‘Li-stuffed’ garnet. Awaka *et al.* (2009 ▸) described the crystal structure of pure LLZO at ambient conditions in space group *I*4_1_/*acd*. Even a small amount of Al in the structure (Al-LLZO) stabilizes the cubic garnet symmetry described in space group *I*a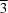 *d* by Geiger *et al.* (2011 ▸). These authors reported that Al could be found on two different tetra­hedral sites using ^27^Al MAS NMR spectroscopy but a final analysis was not possible due to the minor Al content. Rettenwander *et al.* (2014 ▸) reported on ^71^Ga MAS NMR spectroscopy measurements on gallium substituted Li_7-3*x*_Ga_*x*_La_3_Zr_2_O_12_ (Ga–LLZO) indicating a fourfold coordination of the gallium atoms. The authors excluded the presence of Ga at the 24*d* position (*I*a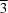 *d*) and assumed that the local symmetry could be lower than indicated by diffraction methods. In principle, the following exchanges are possible: (*i*) 3 Li^+^ ↔ Ga^3+^ + 2 voids, which is the most probable one and yields a good explanation for the higher conductivity due to the higher lithium atom jump probability to empty positions as discussed (Rettenwander *et al.*, 2014 ▸); (*ii*) La^3+^ ↔ Ga^3+^, a valence-neutral exchange which should lead to a dynamical disorder of the gallium atoms in order to lower the coordination number and shorten the Ga—O bond lengths for bond-valence balance, taking the different radii into account. The valence-neutral exchange should finally lead to higher displacement parameters of the atoms on the lanthanum position compared to that of the lighter zirconium atoms. (*iii)* Zr^4+^ ↔ Ga^3+^ + Li^+^, which needs slightly more lithium for charge balance and could therefore be of minor probability.

## Structural commentary   

**Figure 1:**
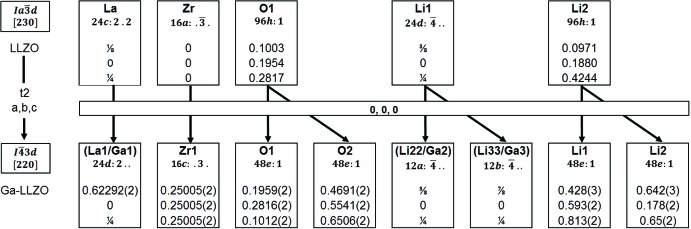
Bärnighausen tree (Bärnighausen, 1980 ▸) of the group–subgroup relation between cubic LLZO and the symmetry-reduced cubic Ga–LLZO.

**Figure 2:**
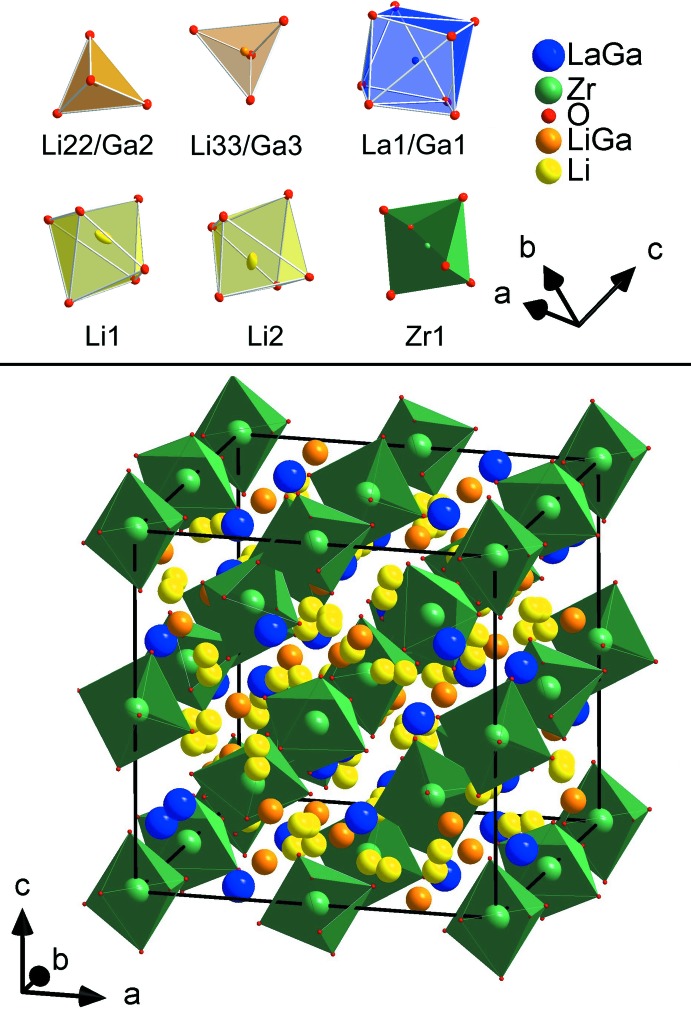
Crystal structure of Li_6.43(2)_Ga_0.52(3)_La_2.67(4)_Zr_2_O_12_ (Ga–LLZO) with all possible atom positions between the ZrO_6_ octa­hedra (bottom) and the atom position specific coordination polyhedra (top). Displacement ellipsoids (top) are given at the 50% probability level.

The unit cell of the obtained single crystals could be well indexed using a body-centered cubic lattice with lattice parameter *a* = 12.9681 (15) Å. The space group determination with *XPREP* (Bruker, 2014 ▸) leads at once to the highest possible space group *I*a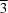 *d*. However, a satisfactory structure solution or refinement with published structural data (Geiger *et al.*, 2011 ▸) in this space group type was not possible. Consequently, structure solutions by charge flipping (Bruker, 2009 ▸) were tried in all possible subgroups of *I*a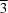 *d* and the lowest *R*-values were obtained for the charge-flipping run in space group *I* 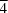
3*d*. Subsequent refinements lead to the present structure model and clearly indicate the substitution of Ga^3+^ on the former 24*c* La^3+^ site as well as 24*d* Li^+^ site in the aristotype in space group *I*a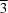 *d*. The latter site splits into two sites due to the symmetry reduction as indicated by the Bärnighausen tree (Bärnig­hausen, 1980 ▸) given in Fig. 1 ▸. The deviation from six symmetry-equivalent Zr—O distances in LLZO (*I*a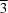 *d*) results in a distortion of the ZrO_6_ octa­hedron with Zr—O distances of 3 × 2.095 (2) and 3 × 2.113 (2) Å in Ga–LLZO. Another significant reduction of the highest possible symmetry for LLZO is the distortion of the eightfold coordinate La position (Fig. 2 ▸), for which distances between 2.496 (2) and 2.595 (2) Å are found in Ga–LLZO. This distortion results from the splitting of the 96*h* position of the oxygen atom in *I*a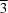 *d* into two 48*e* positions in *I* 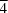
3*d* (Fig. 1 ▸). Because the two lithium positions (Li22 and Li32) occupied by gallium are in principle identical to those positions of the higher symmetry structure (but with slightly shorter bond length due to the gallium substitution, *viz*. 4 × 1.916 (1) Å in LLZO and 4 × 1.908 (2) Å in Ga–LLZO), and the Li1 and Li2 positions are not occupied by gallium, the symmetry reduction is a confirmation of gallium atoms to be found also on the lanthanum position. This is also supported by the higher displacement parameter of the La site compared to the Zr site, as explained previously.

## Synthesis and crystallization   

The synthesis was configured to yield a compound with nominal composition Li_6.25_Ga_0.25_La_3_Zr_2_O_12_. 2 g of a stoichiometric mixture of the pre-dried (30 h at 373 K in vacuum) educts Li_2_O (with an excess of 10%_wt_ to compensate the lithium loss due to thermal treatment), La_2_O_3_, ZrO_2_ and Ga_2_O_3_ was weighted into a WC milling beaker (45 ml, 100 WC milling balls of 5 mm diameter, Fritsch, Germany) under inert conditions (glovebox) and high-energy ball-milled in a planetary ball mill (Pulverisette 7 *premium line*, Fritsch, Germany) under argon atmosphere for 8 h at a rotational speed of 10 s^−1^ as reported previously (Düvel *et al.*, 2012 ▸) for Al-substituted LLZO. The obtained powder was pressed to a pellet using a uniaxial pressure of 0.8 GPa. A stack of three pellets was placed on a platinum ring seated on a corundum plate, covered with a corundum crucible and heated for 12 h at 1323 K in a muffle furnace before cooling to room-temperature. The middle pellet from the stack had smooth green color and showed visible grains. The surface of the pellet was grey and brittle and consisted mainly of lanthanum zirconates due to Li loss. From this pellet single crystals were extracted using a polarization microscope. Rietveld refinement of X-ray powder diffraction data of the green product shows a mixture of 96.8 (9)%_wt_ cubic garnet-type Ga–LLZO and 3.2 (9)%_wt_ Li_2_ZrO_3_ with a lattice parameter of *a* = 12.9738 (19) Å for its garnet-type structure. Energy dispersive X-ray analysis of the single crystal gave a tentative formula of Li_6.5 (1)_Ga_0.5 (1)_La_2.8 (1)_Zr_2.0 (1)_O_12_, in good agreement with the refined formula Li_6.43 (2)_Ga_0.52 (3)_La_2.67 (4)_Zr_2_O_12_ determined from single crystal X-ray diffraction data.

## Refinement   

Crystal data, data collection and structure refinement details are summarized in Table 1 ▸. Structure refinement was carried out as a two-component (merohedral) twin. Sites showing a statistical occupancy were constrained with respect to positions and anisotropic displacement parameters. An independent refinement of the anisotropic displacement parameters of Ga and Li on the Ga2/Li22 and Ga3/Li33 sites was not possible, although the reflection-to-parameter ratio is rather high. To ensure charge neutrality during the refinement of the Ga and Li occupancies on the Ga2/Li22 and Ga3/Li33 sites, the occupancies were restrained to exchange three Li atoms against one Ga atom.

## Supplementary Material

Crystal structure: contains datablock(s) I. DOI: 10.1107/S2056989016001924/wm5261sup1.cif

Structure factors: contains datablock(s) I. DOI: 10.1107/S2056989016001924/wm5261Isup2.hkl

CCDC reference: 1451178

Additional supporting information:  crystallographic information; 3D view; checkCIF report

## Figures and Tables

**Table 1:** Experimental details

Crystal data	
Chemical formula	Li_6.43_Ga_0.52_La_2.67_Zr_2_O_12_
*M*_r_	826.20
Crystal system, space group	Cubic, *I* 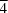 3*d*
Temperature (K)	301
*a* (Å)	12.9681 (15)
*V* (Å^3^)	2180.9 (8)
*Z*	8
Radiation type	Mo *K*α
μ (mm^−1^)	13.41
Crystal size (mm)	0.25 × 0.15 × 0.13

Data collection	
Diffractometer	Bruker APEXII CCD
Absorption correction	Multi-scan (*SADABS*; Bruker, 2014 ▸)
*T*_min_, *T*_max_	0.495, 0.754
No. of measured, independent and observed [*I* > 2σ(*I*)] reflections	472450, 3678, 3508
*R*_int_	0.046
(sin θ/λ)_max_ (Å^−1^)	1.340

Refinement	
*R*[*F* ^2^ > 2σ(*F* ^2^)], *wR*(*F* ^2^), *S*	0.026, 0.056, 1.46
No. of reflections	3678
No. of parameters	50
No. of restraints	3
Δρ_max_, Δρ_min_ (e Å^−3^)	2.08, −1.91
Absolute structure	Flack *x* determined using 1493 quotients [(*I* ^+^)−(*I* ^−^)]/[(*I* ^+^)+(*I* ^−^)] (Parsons *et al.*, 2013 ▸)
Absolute structure parameter	0.045 (9)

Computer programs: *APEX2* and *SAINT* (Bruker, 2014 ▸), *TOPAS* (Bruker, 2009 ▸), *SHELXL2014* (Sheldrick, 2015 ▸), *DIAMOND* (Brandenburg, 1999 ▸) and *publCIF* (Westrip, 2010 ▸).

## supplementary crystallographic information

### Crystal data

**Table:**

Li_6.43_Ga_0.52_La_2.67_Zr_2_O_12_	Mo *K*α radiation, λ = 0.71073 Å
*M**_r_* = 826.20	Cell parameters from 9913 reflections
Cubic, *I*43*d*	θ = 3.1–72.3°
*a* = 12.9681 (15) Å	µ = 13.41 mm^−^^1^
*V* = 2180.9 (8) Å^3^	*T* = 301 K
*Z* = 8	Irregular, green
*F*(000) = 340	0.25 × 0.15 × 0.13 mm
*D*_x_ = 5.033 Mg m^−^^3^	

### Data collection

**Table:**

Bruker APEXII CCD diffractometer	3508 reflections with *I* > 2σ(*I*)
φ and ω scans	*R*_int_ = 0.046
Absorption correction: multi-scan (*SADABS*; Bruker, 2014)	θ_max_ = 72.3°, θ_min_ = 2.2°
*T*_min_ = 0.495, *T*_max_ = 0.754	*h* = −34→34
472450 measured reflections	*k* = −34→34
3678 independent reflections	*l* = −34→34

### Refinement

**Table:**

Refinement on *F*^2^	*w* = 1/[σ^2^(*F*_o_^2^) + (0.011*P*)^2^ + 8.6617*P*] where *P* = (*F*_o_^2^ + 2*F*_c_^2^)/3
Least-squares matrix: full	(Δ/σ)_max_ = 0.049
*R*[*F*^2^ > 2σ(*F*^2^)] = 0.026	Δρ_max_ = 2.08 e Å^−^^3^
*wR*(*F*^2^) = 0.056	Δρ_min_ = −1.91 e Å^−^^3^
*S* = 1.46	Extinction correction: *SHELXL2014* (Sheldrick, 2015), Fc^*^=kFc[1+0.001xFc^2^λ^3^/sin(2θ)]^-1/4^
3678 reflections	Absolute structure: Flack *x* determined using 1493 quotients [(*I*^+^)-(*I*^-^)]/[(*I*^+^)+(*I*^-^)] (Parsons *et al.*, 2013)
50 parameters	Absolute structure parameter: 0.045 (9)
3 restraints	

### Special details

**Table:**

Geometry. All e.s.d.'s (except the e.s.d. in the dihedral angle between two l.s. planes) are estimated using the full covariance matrix. The cell e.s.d.'s are taken into account individually in the estimation of e.s.d.'s in distances, angles and torsion angles; correlations between e.s.d.'s in cell parameters are only used when they are defined by crystal symmetry. An approximate (isotropic) treatment of cell e.s.d.'s is used for estimating e.s.d.'s involving l.s. planes.
Refinement. Refined as a 2-component twin.

### Fractional atomic coordinates and isotropic or equivalent isotropic displacement parameters (Å^2^)

**Table:**

	*x*	*y*	*z*	*U*_iso_*/*U*_eq_	Occ. (<1)
La1	0.62292 (2)	0.0000	0.2500	0.00746 (2)	0.889 (4)
Ga1	0.62292 (2)	0.0000	0.2500	0.00746 (2)	0.111 (4)
Zr1	0.25005 (2)	0.25005 (2)	0.25005 (2)	0.00552 (4)	
O1	0.19586 (18)	0.28163 (18)	0.10116 (16)	0.0118 (3)	
O2	0.46910 (17)	0.55410 (17)	0.65064 (16)	0.0114 (2)	
Li1	0.428 (3)	0.593 (2)	0.813 (2)	0.029 (5)	0.33333 (14)
Li2	0.642 (3)	0.178 (2)	0.065 (2)	0.029 (5)	0.33333 (14)
Ga2	0.3750	0.5000	0.7500	0.0061 (4)	0.0857 (12)
Li22	0.3750	0.5000	0.7500	0.0061 (4)	0.743 (4)
Ga3	0.2500	0.3750	0.0000	0.0110 (9)	0.0403 (12)
Li32	0.2500	0.3750	0.0000	0.0110 (9)	0.879 (4)

### Atomic displacement parameters (Å^2^)

**Table:**

	*U*^11^	*U*^22^	*U*^33^	*U*^12^	*U*^13^	*U*^23^
La1	0.00912 (4)	0.00656 (5)	0.00668 (5)	0.000	0.000	−0.00141 (3)
Ga1	0.00912 (4)	0.00656 (5)	0.00668 (5)	0.000	0.000	−0.00141 (3)
Zr1	0.00552 (4)	0.00552 (4)	0.00552 (4)	0.00021 (3)	0.00021 (3)	0.00021 (3)
O1	0.0129 (6)	0.0138 (6)	0.0087 (5)	0.0014 (5)	−0.0007 (5)	0.0006 (5)
O2	0.0131 (6)	0.0116 (6)	0.0096 (5)	0.0010 (5)	0.0000 (5)	−0.0013 (4)
Li1	0.051 (15)	0.015 (6)	0.022 (6)	−0.013 (9)	−0.013 (8)	0.006 (6)
Li2	0.051 (15)	0.015 (6)	0.022 (6)	−0.013 (9)	−0.013 (8)	0.006 (6)
Ga2	0.0078 (10)	0.0052 (6)	0.0052 (6)	0.000	0.000	0.000
Li22	0.0078 (10)	0.0052 (6)	0.0052 (6)	0.000	0.000	0.000
Ga3	0.0100 (14)	0.013 (2)	0.0100 (14)	0.000	0.000	0.000
Li32	0.0100 (14)	0.013 (2)	0.0100 (14)	0.000	0.000	0.000

### Geometric parameters (Å, º)

**Table:**

La1—O1^i^	2.496 (2)	O2—Li2^xxiii^	2.09 (3)
La1—O1^ii^	2.496 (2)	O2—Zr1^xxiv^	2.113 (2)
La1—O2^iii^	2.520 (2)	O2—Li1	2.23 (4)
La1—O2^iv^	2.520 (2)	O2—La1^xxv^	2.520 (2)
La1—O2^v^	2.590 (2)	O2—Li2^xxvi^	2.54 (3)
La1—O2^vi^	2.590 (2)	O2—La1^xxiii^	2.590 (2)
La1—O1^vii^	2.595 (2)	O2—Li2^xxvii^	2.61 (4)
La1—O1^viii^	2.595 (2)	Li1—O2^xxviii^	1.91 (4)
Zr1—O1^ix^	2.095 (2)	Li1—O2^xxii^	2.04 (3)
Zr1—O1^x^	2.095 (2)	Li1—O1^xxix^	2.16 (3)
Zr1—O1	2.095 (2)	Li1—O1^xxx^	2.65 (3)
Zr1—O2^xi^	2.113 (2)	Li2—O1^viii^	1.90 (3)
Zr1—O2^xii^	2.113 (2)	Li2—O2^vi^	2.09 (3)
Zr1—O2^xiii^	2.113 (2)	Li2—O1^xvi^	2.22 (4)
O1—Li2^xiv^	1.90 (3)	Li2—O1^xxxi^	2.32 (3)
O1—Ga3	1.918 (2)	Li2—O2^xxxii^	2.54 (3)
O1—Li1^xv^	2.16 (3)	Li2—O2^xxxiii^	2.61 (4)
O1—Li2^xvi^	2.22 (4)	Li2—Li2^xxxiv^	2.68 (6)
O1—Li2^xvii^	2.32 (3)	Ga2—O2^xxi^	1.908 (2)
O1—La1^xviii^	2.496 (2)	Ga2—O2^xxii^	1.908 (2)
O1—La1^xix^	2.595 (2)	Ga2—O2^xxviii^	1.908 (2)
O1—Li1^xx^	2.65 (3)	Ga3—O1^xxxv^	1.918 (2)
O2—Ga2	1.908 (2)	Ga3—O1^xxxvi^	1.918 (2)
O2—Li1^xxi^	1.91 (4)	Ga3—O1^xxxvii^	1.918 (2)
O2—Li1^xxii^	2.04 (3)		
			
O1^i^—La1—O1^ii^	73.18 (10)	La1^xviii^—O1—Li1^xx^	79.5 (6)
O1^i^—La1—O2^iii^	160.55 (5)	La1^xix^—O1—Li1^xx^	68.9 (8)
O1^ii^—La1—O2^iii^	111.27 (5)	Ga2—O2—Li1^xxi^	49.8 (10)
O1^i^—La1—O2^iv^	111.27 (5)	Ga2—O2—Li1^xxii^	48.0 (8)
O1^ii^—La1—O2^iv^	160.55 (5)	Li1^xxi^—O2—Li1^xxii^	77.4 (11)
O2^iii^—La1—O2^iv^	71.21 (10)	Ga2—O2—Li2^xxiii^	66.9 (11)
O1^i^—La1—O2^v^	73.18 (7)	Li1^xxi^—O2—Li2^xxiii^	18.2 (10)
O1^ii^—La1—O2^v^	95.73 (8)	Li1^xxii^—O2—Li2^xxiii^	85.1 (16)
O2^iii^—La1—O2^v^	123.67 (5)	Ga2—O2—Zr1^xxiv^	128.57 (12)
O2^iv^—La1—O2^v^	68.75 (9)	Li1^xxi^—O2—Zr1^xxiv^	104.4 (9)
O1^i^—La1—O2^vi^	95.73 (8)	Li1^xxii^—O2—Zr1^xxiv^	87.4 (8)
O1^ii^—La1—O2^vi^	73.18 (7)	Li2^xxiii^—O2—Zr1^xxiv^	88.5 (10)
O2^iii^—La1—O2^vi^	68.75 (9)	Ga2—O2—Li1	44.9 (8)
O2^iv^—La1—O2^vi^	123.67 (5)	Li1^xxi^—O2—Li1	72.9 (12)
O2^v^—La1—O2^vi^	166.44 (9)	Li1^xxii^—O2—Li1	85.9 (12)
O1^i^—La1—O1^vii^	125.03 (5)	Li2^xxiii^—O2—Li1	89.7 (13)
O1^ii^—La1—O1^vii^	68.53 (9)	Zr1^xxiv^—O2—Li1	173.2 (7)
O2^iii^—La1—O1^vii^	72.72 (6)	Ga2—O2—La1^xxv^	94.15 (8)
O2^iv^—La1—O1^vii^	94.96 (7)	Li1^xxi^—O2—La1^xxv^	143.6 (9)
O2^v^—La1—O1^vii^	73.07 (5)	Li1^xxii^—O2—La1^xxv^	80.5 (11)
O2^vi^—La1—O1^vii^	108.77 (5)	Li2^xxiii^—O2—La1^xxv^	161.0 (11)
O1^i^—La1—O1^viii^	68.53 (9)	Zr1^xxiv^—O2—La1^xxv^	103.05 (9)
O1^ii^—La1—O1^viii^	125.03 (5)	Li1—O2—La1^xxv^	77.0 (9)
O2^iii^—La1—O1^viii^	94.96 (7)	Ga2—O2—Li2^xxvi^	57.6 (8)
O2^iv^—La1—O1^viii^	72.72 (6)	Li1^xxi^—O2—Li2^xxvi^	76.3 (11)
O2^v^—La1—O1^viii^	108.77 (5)	Li1^xxii^—O2—Li2^xxvi^	99.9 (14)
O2^vi^—La1—O1^viii^	73.07 (5)	Li2^xxiii^—O2—Li2^xxvi^	91.0 (11)
O1^vii^—La1—O1^viii^	165.12 (9)	Zr1^xxiv^—O2—Li2^xxvi^	172.6 (9)
O1^ix^—Zr1—O1^x^	86.37 (10)	Li1—O2—Li2^xxvi^	14.0 (8)
O1^ix^—Zr1—O1	86.37 (10)	La1^xxv^—O2—Li2^xxvi^	79.4 (7)
O1^x^—Zr1—O1	86.37 (10)	Ga2—O2—La1^xxiii^	122.63 (10)
O1^ix^—Zr1—O2^xi^	93.51 (9)	Li1^xxi^—O2—La1^xxiii^	95.8 (10)
O1^x^—Zr1—O2^xi^	179.59 (11)	Li1^xxii^—O2—La1^xxiii^	170.6 (8)
O1—Zr1—O2^xi^	94.02 (9)	Li2^xxiii^—O2—La1^xxiii^	90.4 (8)
O1^ix^—Zr1—O2^xii^	94.02 (9)	Zr1^xxiv^—O2—La1^xxiii^	100.77 (8)
O1^x^—Zr1—O2^xii^	93.51 (9)	Li1—O2—La1^xxiii^	85.8 (7)
O1—Zr1—O2^xii^	179.59 (11)	La1^xxv^—O2—La1^xxiii^	101.98 (8)
O2^xi^—Zr1—O2^xii^	86.10 (9)	Li2^xxvi^—O2—La1^xxiii^	71.8 (9)
O1^ix^—Zr1—O2^xiii^	179.59 (11)	Ga2—O2—Li2^xxvii^	56.1 (7)
O1^x^—Zr1—O2^xiii^	94.02 (9)	Li1^xxi^—O2—Li2^xxvii^	83.4 (14)
O1—Zr1—O2^xiii^	93.51 (9)	Li1^xxii^—O2—Li2^xxvii^	8.2 (9)
O2^xi^—Zr1—O2^xiii^	86.10 (9)	Li2^xxiii^—O2—Li2^xxvii^	89.2 (9)
O2^xii^—Zr1—O2^xiii^	86.10 (9)	Zr1^xxiv^—O2—Li2^xxvii^	80.4 (7)
Li2^xiv^—O1—Ga3	55.1 (12)	Li1—O2—Li2^xxvii^	93.0 (11)
Li2^xiv^—O1—Zr1	100.2 (12)	La1^xxv^—O2—Li2^xxvii^	78.2 (6)
Ga3—O1—Zr1	129.12 (12)	Li2^xxvi^—O2—Li2^xxvii^	107.0 (12)
Li2^xiv^—O1—Li1^xv^	17.3 (11)	La1^xxiii^—O2—Li2^xxvii^	178.8 (7)
Ga3—O1—Li1^xv^	71.3 (8)	O2^xxviii^—Li1—O2^xxii^	108.5 (14)
Zr1—O1—Li1^xv^	84.8 (8)	O2^xxviii^—Li1—O1^xxix^	98.6 (18)
Li2^xiv^—O1—Li2^xvi^	80.8 (13)	O2^xxii^—Li1—O1^xxix^	93.9 (12)
Ga3—O1—Li2^xvi^	50.0 (9)	O2^xxviii^—Li1—O2	101.2 (12)
Zr1—O1—Li2^xvi^	85.8 (9)	O2^xxii^—Li1—O2	86.8 (14)
Li1^xv^—O1—Li2^xvi^	87.3 (14)	O1^xxix^—Li1—O2	158.9 (18)
Li2^xiv^—O1—Li2^xvii^	78.1 (15)	O2^xxviii^—Li1—O1^xxx^	144.7 (15)
Ga3—O1—Li2^xvii^	48.2 (10)	O2^xxii^—Li1—O1^xxx^	106.5 (15)
Zr1—O1—Li2^xvii^	177.3 (10)	O1^xxix^—Li1—O1^xxx^	83.1 (9)
Li1^xv^—O1—Li2^xvii^	93.8 (10)	O2—Li1—O1^xxx^	76.4 (12)
Li2^xvi^—O1—Li2^xvii^	91.8 (16)	O1^viii^—Li2—O2^vi^	101.0 (12)
Li2^xiv^—O1—La1^xviii^	147.5 (13)	O1^viii^—Li2—O1^xvi^	102.1 (17)
Ga3—O1—La1^xviii^	92.55 (8)	O2^vi^—Li2—O1^xvi^	91.1 (12)
Zr1—O1—La1^xviii^	103.48 (9)	O1^viii^—Li2—O1^xxxi^	98.5 (14)
Li1^xv^—O1—La1^xviii^	163.4 (9)	O2^vi^—Li2—O1^xxxi^	160.3 (14)
Li2^xvi^—O1—La1^xviii^	79.1 (7)	O1^xvi^—Li2—O1^xxxi^	82.0 (13)
Li2^xvii^—O1—La1^xviii^	77.3 (7)	O1^viii^—Li2—O2^xxxii^	151.8 (19)
Li2^xiv^—O1—La1^xix^	94.7 (9)	O2^vi^—Li2—O2^xxxii^	86.9 (13)
Ga3—O1—La1^xix^	123.13 (10)	O1^xvi^—Li2—O2^xxxii^	104.8 (10)
Zr1—O1—La1^xix^	100.26 (9)	O1^viii^—Li2—O2^xxxiii^	84.1 (12)
Li1^xv^—O1—La1^xix^	89.9 (10)	O2^vi^—Li2—O2^xxxiii^	85.2 (14)
Li2^xvi^—O1—La1^xix^	173.1 (9)	O1^xvi^—Li2—O2^xxxiii^	173.3 (16)
Li2^xvii^—O1—La1^xix^	82.1 (10)	O1^xxxi^—Li2—O2^xxxiii^	99.7 (11)
La1^xviii^—O1—La1^xix^	102.50 (8)	O2^xxxii^—Li2—O2^xxxiii^	69.5 (10)
Li2^xiv^—O1—Li1^xx^	81.4 (12)	O2^xxi^—Ga2—O2^xxii^	114.14 (7)
Ga3—O1—Li1^xx^	60.5 (8)	O2^xxi^—Ga2—O2^xxviii^	100.49 (13)
Zr1—O1—Li1^xx^	169.2 (8)	O2^xxii^—Ga2—O2^xxviii^	114.14 (7)
Li1^xv^—O1—Li1^xx^	95.1 (9)	O1—Ga3—O1^xxxv^	113.48 (7)
Li2^xvi^—O1—Li1^xx^	105.0 (14)	O1—Ga3—O1^xxxvi^	101.73 (14)
Li2^xvii^—O1—Li1^xx^	13.3 (11)	O1^xxxv^—Ga3—O1^xxxvi^	113.48 (7)

Symmetry codes: (i) −*y*+3/4, *x*−1/4, −*z*+1/4; (ii) −*y*+3/4, −*x*+1/4, *z*+1/4; (iii) −*x*+5/4, −*z*+3/4, *y*−1/4; (iv) −*x*+5/4, *z*−3/4, −*y*+3/4; (v) −*z*+5/4, *y*−3/4, −*x*+3/4; (vi) −*z*+5/4, −*y*+3/4, *x*−1/4; (vii) −*z*+3/4, −*y*+1/4, *x*+1/4; (viii) −*z*+3/4, *y*−1/4, −*x*+1/4; (ix) *y*, *z*, *x*; (x) *z*, *x*, *y*; (xi) *z*−1/4, *y*−1/4, *x*−1/4; (xii) *y*−1/4, *x*−1/4, *z*−1/4; (xiii) *x*−1/4, *z*−1/4, *y*−1/4; (xiv) −*z*+1/4, *y*+1/4, −*x*+3/4; (xv) *x*−1/4, −*z*+5/4, −*y*+3/4; (xvi) −*x*+1, −*y*+1/2, *z*; (xvii) *x*−1/2, −*y*+1/2, −*z*; (xviii) −*y*+1/4, −*x*+3/4, *z*−1/4; (xix) *z*−1/4, −*y*+1/4, −*x*+3/4; (xx) *y*−1/2, *z*−1/2, *x*−1/2; (xxi) −*x*+3/4, *z*−1/4, −*y*+5/4; (xxii) *x*, −*y*+1, −*z*+3/2; (xxiii) *z*+1/4, −*y*+3/4, −*x*+5/4; (xxiv) *y*+1/4, *x*+1/4, *z*+1/4; (xxv) −*x*+5/4, *z*+1/4, −*y*+3/4; (xxvi) −*z*+1/2, *x*, −*y*+1; (xxvii) −*z*+1/2, −*x*+1, *y*+1/2; (xxviii) −*x*+3/4, −*z*+5/4, *y*+1/4; (xxix) *x*+1/4, −*z*+3/4, −*y*+5/4; (xxx) *z*+1/2, *x*+1/2, *y*+1/2; (xxxi) *x*+1/2, −*y*+1/2, −*z*; (xxxii) *y*, −*z*+1, −*x*+1/2; (xxxiii) −*y*+1, *z*−1/2, −*x*+1/2; (xxxiv) *z*+3/4, −*y*+1/4, −*x*+3/4; (xxxv) *z*+1/4, −*y*+3/4, −*x*+1/4; (xxxvi) −*x*+1/2, *y*, −*z*; (xxxvii) −*z*+1/4, −*y*+3/4, *x*−1/4.
